# State of the Art and Future Opportunities in MRI-Guided Robot-Assisted Surgery and Interventions

**DOI:** 10.1109/jproc.2022.3169146

**Published:** 2022-05-03

**Authors:** Hao Su, Ka-Wai Kwok, Kevin Cleary, Iulian Iordachita, M. Cenk Cavusoglu, Jaydev P. Desai, Gregory S. Fischer

**Affiliations:** Department of Mechanical and Aerospace Engineering, North Carolina State University, Raleigh, NC 27695 USA; Department of Mechanical Engineering, The University of Hong Kong, Hong Kong; Children’s National Health System, Washington, DC 20010 USA; Laboratory for Computational Sensing and Robotics (LCSR), Johns Hopkins University, Baltimore, MD 21218 USA; Department of Electrical, Computer, and Systems Engineering, Case Western Reserve University, Cleveland, OH 44106 USA; Wallace H. Coulter Department of Biomedical Engineering, Georgia Institute of Technology, Atlanta, GA 30332 USA; Department of Robotics Engineering, Worcester Polytechnic Institute, Worcester, MA 01609 USA

**Keywords:** Fiber optic sensors, image-guided surgery, magnetic resonance imaging (MRI)-compatible robots, piezoelectric actuators, sensors and actuators, surgical robots

## Abstract

Magnetic resonance imaging (MRI) can provide high-quality 3-D visualization of target anatomy, surrounding tissue, and instrumentation, but there are significant challenges in harnessing it for effectively guiding interventional procedures. Challenges include the strong static magnetic field, rapidly switching magnetic field gradients, high-power radio frequency pulses, sensitivity to electrical noise, and constrained space to operate within the bore of the scanner. MRI has a number of advantages over other medical imaging modalities, including no ionizing radiation, excellent soft-tissue contrast that allows for visualization of tumors and other features that are not readily visible by other modalities, true 3-D imaging capabilities, including the ability to image arbitrary scan plane geometry or perform volumetric imaging, and capability for multimodality sensing, including diffusion, dynamic contrast, blood flow, blood oxygenation, temperature, and tracking of biomarkers. The use of robotic assistants within the MRI bore, alongside the patient during imaging, enables intraoperative MR imaging (iMRI) to guide a surgical intervention in a closed-loop fashion that can include tracking of tissue deformation and target motion, localization of instrumentation, and monitoring of therapy delivery. With the ever-expanding clinical use of MRI, MRI-compatible robotic systems have been heralded as a new approach to assist interventional procedures to allow physicians to treat patients more accurately and effectively. Deploying robotic systems inside the bore synergizes the visual capability of MRI and the manipulation capability of robotic assistance, resulting in a closed-loop surgery architecture. This article details the challenges and history of robotic systems intended to operate in an MRI environment and outlines promising clinical applications and associated state-of-the-art MRI-compatible robotic systems and technology for making this possible.

## I. INTRODUCTION

The first magnetic resonance imaging (MRI)-guided intervention was performed by Jolesz [1] for tumor resection neurosurgery at the Harvard Medical School in 1993. Ever since the first MRI-guided robot, a 6-degrees of freedom (DOFs) manipulator actuated with ultrasonic motors developed by Masamune for stereotactic neurosurgery inside a 0.5-T scanner in 1995 [2], the field of MRI-guided robot-assisted intervention has gained momentum to translate engineering innovation to improved benefits of clinical care. The overarching goal of image-guided robotic assistance is to improve clinical outcomes through more accurate, less invasive, and more effective interventions taking into account medical imaging-based feedback.

From the first principal perspective, MRI has unique advantages over other medical imaging modalities, such as computed tomography (CT) and ultrasound. It is an excellent guidance tool for interventional procedures that incorporate robotic assistance. Unique features of MRI-guided interventions include high spatial resolution, excellent soft-tissue contrast, real-time imaging of and arbitrary scan plane geometry for continuous intraoperative tracking of surgical tools, on-the-fly adjustment of the imaging plane, multiparameter imaging (e.g., MR thermal imaging (MRTI) or thermometry, functional MRI (fMRI), diffusion, and dynamic contrast), and no ionizing radiation hazard to patients and clinicians as would occur when using CT or X-ray fluoroscopy.

The advantages of MRI-compatible robots are capitalized on their ability for precision control of position/velocity and forces/torques and intraoperative feedback from 3-D image guidance. The use of robotic assistants within the MRI bore enables intraoperative MR imaging (iMRI) to guide a surgical intervention in a closed-loop fashion by tracking anatomy, instrumentation, and therapy delivery and using that information to guide the robotic platform. This is in contrast to the use of static preoperative imaging in a typical operating room (OR) setting, fusing preoperative MRI to another intraoperative modality, such as ultrasound, or having to repeatedly move a patient out of the bore for intervention and back into the bore for imaging confirmation. However, MRI imposes unique challenges for mechanical design, control electronics, actuation, sensing, and closed-loop control of robotic systems, including electromagnetic interference (EMI), material incompatibility, and confined space inside the MRI bore. Thus, MRI-compatible robotic systems often cannot be constructed using “off-the-shelf” robotics components, making the designs more custom, complex, and costly than other imaging modalities, which is a key design consideration. For context, the term MRI-compatible is used here to refer to robots and devices specifically designed to be used within the vicinity of the MRI scanner and is compatible with the MRI environment. As such, to be compatible with the MRI environment, these devices must be safe to operate within their intended use cases, their functionality should not be negatively impacted by the MRI, and they should not detrimentally affect MR image quality in a clinically significant way.

Since the review paper, *MR robotics: a critical tool for image-guided interventions, clinical diagnostics and neuroscience* by Gassert, Burdet, and Chinzei published in *IEEE Engineering in Medicine and Biology Magazine* in 2008, tremendous advances have been made in MRI-compatible robotics, ranging from enabling technologies and advanced system development to clinical evaluations. Those systems largely aim to synergize the imaging capability of MRI and the manipulation capability of robots, formulating the “closed-loop intervention.” This article focuses on the major accomplishments in the last two decades of MRI-guided robotic systems to assist surgery and interventions, and provides a retrospective assessment of the field. Though MRI-compatible robots also play a unique role in neuroimaging and neurorehabilitation, this is not the focus of this article. More details about robots for rehabilitation can be found in [3]. In addition, a survey of some other recent advances in MRI-guided surgical robots can be found in [4]-[6].

This article is intended to outline and present examples of the current state of the art, and associated trends and opportunities in MRI-guided robots. The focus of this article is on providing insight into the field of MRI-compatible robotics, rather than providing a comprehensive review of all related research and commercial work. The primary goals of this article are threefold: 1) to summarize representative work about MRI-compatible robots for interventional applications; 2) to identify enabling technologies (e.g., actuators, sensors, and closed-loop control) for MRI-compatible robots; and 3) to provide future perspectives about the limitations, open questions, and challenges of the current research landscape.

### A. Benefits of Intraoperative MRI-Guided Robot-Assisted Surgery and Interventions

There are well-established advantages to using medical imaging to guide surgical interventions. A description of various such cases was prepared by a Society of Interventional Radiology research consensus panel and is discussed in [7]. Utilizing MRI to guide interventions offers unique advantages for soft-tissue visualization over CT and ultrasound because MRI provides high-fidelity soft-tissue contrast and spatial resolution. In addition, MRI provides simultaneous imaging of both soft tissue and intervention instruments (e.g., needles and catheters), thus enabling one to interactively adjust and control the interventional plan. Furthermore, MRI, as a multiparametric imaging method, provides the sensing capability to a wide variety of physiological signals, including oxygenation, flow, temperature, strain, and so on.

One key benefit of MRI-guided robot-assisted interventions is their potential to increase accuracy. Robotic assistance could improve positioning accuracy and reproducibility due to closed-loop control and coordinated multiple-axis motion. Specifically, intraoperative MRI can track tissue motion and instrument deflection to ensure that the surgical plan is executed as intended. Imaging can facilitate instrument tracking and motion compensation to dynamically adjust intervention strategies. As a motivating example, Hatiboglu *et al*. [8] found that, in over 40% of reported glioma surgery cases, the surgeons readjust their surgical approach based on updated information from intraoperative MRI.

Another benefit of MRI-guided robot-assisted interventions is reduced procedure time. Standard intraoperative procedures have to move the patient out of the bore for intervention and back into the bore for imaging. As appropriately designed robots can perform interventions inside the MRI bore, simultaneous intervention and imaging could reduce procedure time while improving outcomes. As investigated for prostate biopsy (Bx), a 2-DOF Cartesian-type robot [9] was able to increase accuracy and reduce the mean core procedure time from 100.6 (56 patients using manual procedures) to 90.8 min (43 patients using robotic assistance). Another example was provided by Li *et al*. [10], which describes significant time savings when using an MRI-compatible robot for neurosurgery, in particular for bilateral placement of leads for deep brain stimulation (DBS). In this scenario, not only can MRI enable updating the plan based on brain shift, but the high spatial resolution allows image-guided confirmation of targeting and can eliminate the need for very time-consuming microelectrode recording (MER).

Furthermore, MRI-guided robot-assisted interventions can significantly improve ergonomics. Manual interventions in the closed-bore scanner are ergonomically challenging and, in some cases, infeasible. A typical closed-bore MRI scanner has a bore diameter of 60–70 cm in diameter, and it takes 75–90 cm to reach the isocenter of the scanner. In addition, it is difficult to see intraoperative iMRI when reaching into the bore. While more low field open scanners have been introduced for interventional procedures, their field strength and image quality do not match that of a modern high field closed-bore diagnostic scanner. Fernández-Gutiérrez *et al*. [197] compared ergonomic procedural differences between fluoroscopy-guided and MRI-guided procedures. It was noted that, without assistance, clinicians tended to maintain ergonomically disadvantageous postures while carrying out the procedures under MRI, in comparison with the performance in the angiography suite. The use of robotics can significantly improve the ergonomics of performing procedures inside the MRI scanner. Robots can remain in the bore for a sustained amount of time, while human operators can perform interventions *via* teleoperation of robots that are inside MRI. Furthermore, robots can perform precise interventions within the bore, while the clinician is comfortably outside the bore (either in the scanner room or in the adjacent console room area).

### B. Design Requirements of Robots for Intraoperative MRI-Guided Surgery and Interventions

Challenges for robot-assisted MRI-guided interventions arise from electromagnetic compatibility due to the MRI environment and space constraints due to typical closed-bore geometry. Bidirectional MRI compatibility requires that neither the device affects the scanner function (e.g., image artifacts) nor the scanner affects the device function. With regard to the safety of devices used in the MRI environment, three categories of MRI device safety adopted by the U.S. Food and Drug Administration (FDA) are “MR Safe,” “MR Conditional,” and “MR Unsafe.” FDA follows the classification (ASTM F2503) by the American Society for Testing and Materials (ASTM). Though this FDA guidance classifying safety does not use the term “MRI-compatible,” as noted earlier, this term is referring to devices intended to operate in the MRI environment. In particular, it is a commonly used and meaningful description, especially in an engineering context to describe device functionality and its effect on MRI image quality, in addition to safety. An “MRI-compatible” device should not introduce unanticipated safety hazards, should not have its intended functions deteriorated by the MRI system, and should not significantly affect imaging quality.

The above-noted ASTM F2503 standard defines the three possible safety classifications for medical devices used in MRI environments: MR Safe, MR Conditional, and MR Unsafe. The term “MR Safe” requires that the device and any associated components inside the MRI scanner room contain no electronics, metallic, or other conductive components and are inherently safe. It should be noted that this is a specific classification but does not imply that other devices are not safe for use in MRI. For example, patient monitoring systems, anesthesia equipment, injectors, needles, other instruments, and imaging coils designed to operate in MRI all contain conductive material and, by this definition, would not be classified as MR Safe. Rather, many devices are classified as “MR Conditional,” which means that the device is safe for use in MRI under the appropriately validated conditions in which it is intended to operate (i.e., the device poses no known hazards in a specified environment with specified conditions of use). The “MR Unsafe” classification is designated to items that pose risks to the patient, medical personnel, or other persons within the MR environment and should not be brought into the MRI room.

Certain sensing and actuation technologies can be made to be MR Safe (e.g., specially designed pneumatic actuators and fiberoptic sensors) and, when combined with nonconductive mechanisms, can be considered inherently safe. However, thoughtfully designed devices for MRI can, and often do, incorporate electronics and other conductive materials safely and effectively. Each sensing and actuation approach has its own pros and cons, and the optimal approach is driven by the clinical and engineering requirements for a particular application. Additional standards are also used to assess devices intended for use inside the MRI scanner. ASTM F2052 provides a test method for the measurement of magnetically induced displacement force. ASTM F2213 provides standard methods for measuring magnetically induced torque for medical devices in the region of the uniform magnetic field in an MR system. ASTM F2182 provides a method for the measurement of RF-induced heating. ISO/TS 10974 provides a test method for the assessment of gradient-induced vibration for active implantable medical devices (AIMDs). The tests outlined in ISO/TS 10974 measure the amount of unintended charge and the current flow due to time-varying gradient magnetic fields. The various approaches for sensing, actuation, and control described in this article can be safe for use in the MR environment when appropriately designed and validated to do so.

An MRI scanner may affect the function of a robot because the static magnetic field can generate torque/force to a device made of ferromagnetic materials or to conductive materials moving in the scanner due to induced eddy currents. Switching magnetic gradients and radio frequency (RF) pulses of the scanner can induce RF interference, leading to EMI, vibration, and other adverse effects. Moving conductive materials or switching magnetic field gradients could result in eddy currents that may cause thermal, mechanical effects, image distortion, or RF burns in the patient.

Robotic components may affect iMRI because of materials and electronics. Ferromagnetic material typically results in significant image distortion, while nonferromagnetic conductors could also induce field distortion and susceptibility artifacts. Electric currents of a robot could induce RF emissions that are picked up as signal noise on the MRI scanner’s receive coils. Thus, significant engineering effort is necessary when designing a system intended to operate inside the bore of MRI scanners since conventional robot components, including materials, actuators, and sensors, typically cannot be employed.

As with all surgical robots, in addition to the MRI-specific design considerations, MRI-compatible robots must address safety issues in terms of software control and redundancy, as described in [198] and [199]. Fei *et al*. [198] created a safety model that uses three axes: software, hardware, and control policy to analyze safety factors. The policy was defined as hazard identification and safety insurance control that include seven principles [198]. The hazard identification and safety assurance control strategies were adopted for safety enhancement in mechanical, electrical, and software design. Medical manipulators are usually equipped with redundant position encoders and ways to mechanically limit the speed and/or force that the robot can exert. If a consistency check failure is detected, two common approaches are to freeze robot motion or to cause the manipulator to go limp—the selection is very application dependent and, in some cases, may vary for different DOFs of the robot (e.g., a fail-safe state of a robot arm locking in position but allowing passive retraction of an instrument). A unique feature of working within the MRI environment is that the imaging system itself can, in some cases, be used as a primary and/or redundant method of localization and confirmation, as described later in this article.

## II. INTRAOPERATIVE MRI-GUIDED SURGICAL ROBOTS

Intraoperative MRI refers to a scenario in which a physician is able to image the patient *via* an MRI scanner, while the patient is undergoing a surgical procedure in or beside the MRI scanner. iMRI can be performed in specialized interventional MR suites or can be implemented in traditional diagnostic MRI rooms with appropriate considerations. iMRI can generally be classified as either continuous intraoperative imaging while working on the patient within the bore or iterative imaging wherein the patient is translated in and out of the bore between cycles of imaging and performing the surgical intervention. In the former, with robot-assisted procedures, the robot can operate from within the MRI bore and, in many cases, perform simultaneous robotic intervention and imaging. In the latter, robots are intended for interleaved imaging and robotic intervention and typically operate from beside the scanner bore (e.g., NeuroArm robot [21]). Intraoperative robotic MRI-guided interventions are challenging both from the robotics viewpoint and the imaging viewpoint. Broadly speaking, some of the factors that limit the widespread use of robotics in clinical MRI-guided interventions are: 1) the choice of materials used inside the magnet; 2) limited actuators choice; 3) sensors used to localize the robot and the associated hardware for interventions; 4) the speed and quality of the acquired image for real-time control of the robot during the intervention; and 5) finally, the limitations on the available workspace for deploying the robotic system inside the MRI bore. As an example, interventions requiring real-time correction of the needle position in an MRI-guided procedure will potentially require multiple degree-of-freedom robotic systems with sufficient actuator output and the ability to correct the position in real time, especially when the needle is around critical structures. As the number of actuators increases, so does the need for additional sensors and the challenge to maintain image quality without loss of signal-to-noise ratio (SNR) during the procedure. While there have been several advances in the field, clinical implementation of robotic MRI-guided interventions is still a promising area of research to build a complete intervention system for a variety of procedures.

Melzer *et al*. [11] developed the first commercially available MRI-compatible robotic system for percutaneous interventions INNOMOTION that received CE Mark approval. The MRI and CT-compatible system had 5-DOF and was intended for MRI-guided sciatic pain and facet joint treatments, biopsies, drainages, and CT-guided osteosynthesis. Clinical evaluation of safety and accuracy was successfully conducted on 16 patients. This work is an important case study since, in the end, it was not commercially viable and was discontinued.

Design decisions for MRI-guided robots include surgical procedures (how to fit the robot with the patient and the scanner and to define an effective workflow), table-mounted versus body-mounted (to compensate for human movements) robots, active DOF (how many DOF should be actuated), manual DOF (e.g., robots provide needle alignment, while clinician does needle placement), and control methods (automation versus teleoperation versus robot–human cooperative manipulation of surgical tools).

This section reviews examples of robots for several representative interventional procedures to summarize technological development, engineering evaluation, and status of clinical evaluations.

### A. MRI-Guided Robots for Prostate Interventions

Prostate intervention is one of the most active research areas in MRI-guided robotics, in particular, before 2015. Transperineal and transrectal interventions are the mainstream interventional methods. The current gold standard of prostate biopsy is transrectal ultrasound (TRUS) guidance. In the transperineal approach, patients are in a supine position, and a mechanical grid is placed over the perineum to assist the needle placement through the perineum. A TRUS-guided prostate biopsy can present difficulties in visualizing cancerous tissues because of relatively low-quality ultrasound images of the tissue and needle. While TRUS-MRI fusion systems exist, they rely on intraoperative ultrasound and not real-time MRI feedback during a procedure. Therefore, MRI robots have been introduced to enable intraoperative imaging guidance and automatic targeting due to their ability to provide detailed images of anatomical visualization.

D’Amico *et al*. [12] pioneered the method of MRI-guided brachytherapy for prostate cancer in 1997. In 2000, the earliest work for MRI-guided prostate intervention robots was performed by Chinzei *et al*. [13] for open MRI to guide needles during biopsy and brachytherapy. Since then, several MRI-guided robots have been developed, and a few of them reached first-in-human trials.

In 2018, a robotic device for in-bore MRI-guided prostate biopsies (Soteria^1^ Remote Controlled Manipulator, Soteria Medical, Arnhem, The Netherlands) was introduced into practice. It had a pneumatic robotic arm, which provided more freedom of movement, and imaging registration software to directly move the introducer. It also provided a projection of the needle trajectory based on postmovement images to verify the correct orientation toward the target. Soteria received CE certification and FDA registration.

#### 1) Robots for Transrectal Prostate Interventions:

**Fig. 2.**
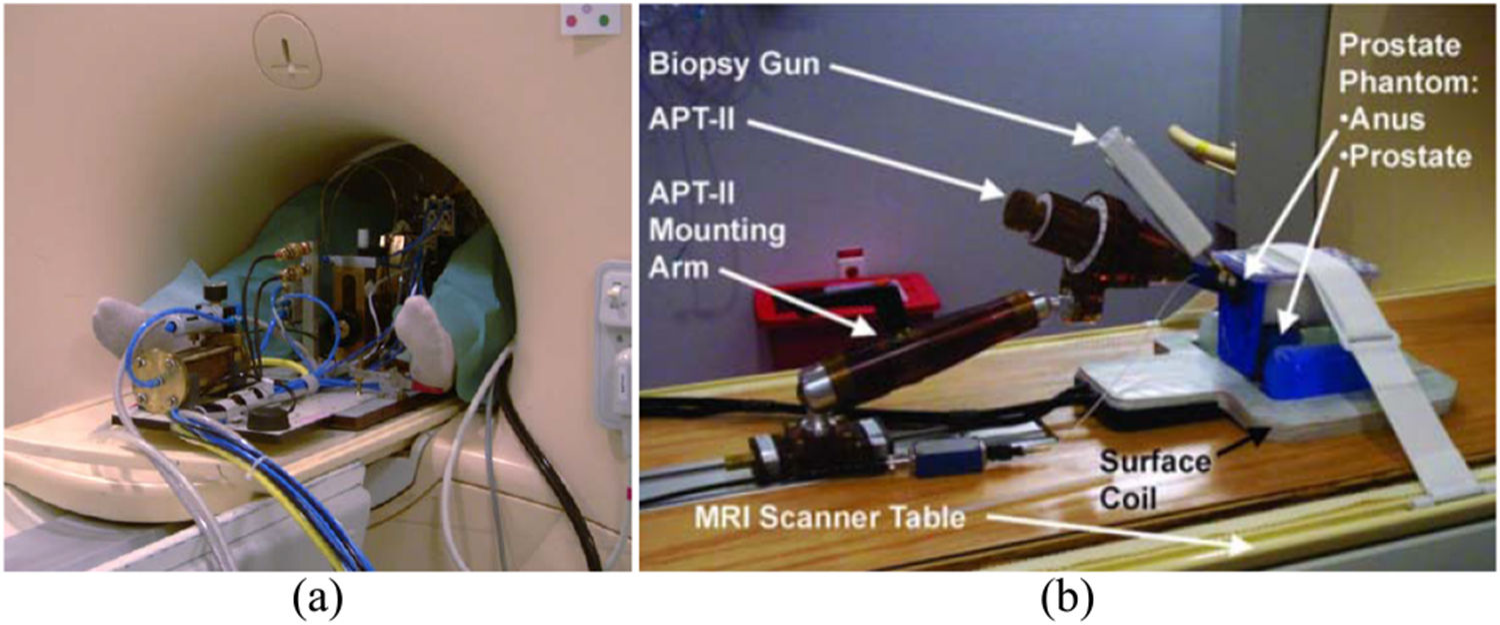
(a) Pneumatically and hydraulically driven robot for transperineal prostate interventions at the University Medical Center Utrecht [16]. (b) MRI-guided transrectal manipulator of the APT II system for transrectal needle access to the prostate was developed at Johns Hopkins University [14].

Transrectal access is well tolerated by patients as it requires only local anesthesia. Workflow adaptation of MRI-guided transrectal interventions is relatively easier because the current standard for prostate biopsy is TRUS, while clinicians usually have extensive experience with transrectal access. Krieger *et al*. [14] reported the first clinical trial of an MRI-compatible robot (no powered actuation) for transrectal prostate biopsy. The system shown in Fig. 2(b) utilized a manipulator employing a steerable needle channel and a 6-DOF hybrid tracking method comprising passive fiducial tracking for initial registration and subsequent motion measurement using fiber optical encoders and mechanical scales. Two clinical human procedures were performed, and both took 30 min. The needle placement accuracy was sufficient to target clinically significant prostate cancer foci (0.5 mL).

Yakar *et al*. [15] presented a 5-DOF robotic needle guide for transrectal prostate biopsy. The needle guide had a suction cup as a safety mechanism, which automatically released when the force to the patients’ rectal wall reached a preset threshold value. A total of 17 cancer-suspicious regions were detected with robotic assistance in ten patients, and the median time of the entire biopsy procedure was 76.5 min (range: 45–105 min). 13 of the 17 cancer-suspicious lesions were targeted with the robot-assisted biopsy, resulting in a cancer detection rate of 56%.

#### 2) Robots for Transperineal Prostate Interventions:

The transperineal method may be advantageous over the transrectal approach to avoid excessive physical damage to the anterior rectal wall. Bosch *et al*. [16] reported the first human trial of transperineal prostate intervention on one patient and the procedure time was 1.5 h. This 5-DOF MRI compatible robot contained a pneumatically driven tapping device to insert a needle with a stylet stepwise into the patient, as shown in Fig. 2(a). When the target was reached, an oncologist manually placed fiducial gold markers (i.e., small seed). This study proved that robot-assisted MRI-guided transperineal needle placement and radiative seed delivery in the prostate were feasible.

Schouten *et al*. [17] further developed the pneumatic robot originally presented in [16] for transperineal biopsies of 13 patients with 32 needle positions under real-time imaging. The mean targeting error for both the robotic and manual approaches was almost similar (5.7 versus 5.8 mm, respectively). The mean time to perform the entire biopsy procedure was 76 min (range: 60–100 min) and 61 min (range: 52–64 min) with the robotic and manual approaches, respectively. Patients were moved out of the scanner bore for manual adjustment and insertion of the biopsy device, which may lead to a higher procedure time. If the robot was designed for teleoperated needle placement, it might be possible to reduce procedure time.

Tilak *et al*. [9] reported a clinical evaluation of a 2-D planar robotic needle guide template for in-bore transperineal MRI-guided prostate biopsy with 96 patients. The robotic approach demonstrated statistically higher accuracy (2.39 mm) compared with the manual approach. Furthermore, the robotic group’s core procedure time was 90.8 min in contrast to 100.6 min of the manual group. Another promising result was that of detecting cancer in 174 of 541 cores (32.16%) in the manual group, whereas 137 of 310 cores (44.19%) were cancer positive in the robotic group. This suggested that the robotic approach had a greater chance to provide a positive cancer core than the manual approach (*p* = 0.018).

A 4-DOF piezoelectrically actuated robot acting as an angulated robotic needle guide for transperineal prostate biopsy was used in a 27-patient clinical trial at the Brigham and Women’s Hospital. The robot design and system architecture are detailed in [18], and an in-depth analysis of the needle-tissue interaction and its effect on needle placement accuracy is described in [19]. One challenge to high clinical accuracy was the presence of deviation resulting from the contact between the needle and the skin. This emphasizes the need for real-time monitoring of the instrument and the target and updating it in real time. The previous system was converted to a 6-DOF robot that allows robotic insertion and rotation of the needle tip to control the path based on the bevel direction, which is described in [20]. This work demonstrates an approach for cooperatively controlled needle insertion with automated trajectory compensation based on real-time iMRI.

### B. MRI-Guided Robots for Neurosurgery

Neurosurgical procedures include a variety of applications, including biopsy, injection of therapeutics, laser surgery, radiation treatment, thermal ablation, and electrode placement. Since the human skull encompasses the brain, applications of ultrasound imaging are typically limited in neurosurgery. CT imaging does not provide high contrast images of the soft tissue of the brain, and it imposes ionizing radiation on patients and clinicians. Thus, MRI is particularly suitable for neurosurgery with the advantage of visualizing both the structure and function of the brain and can monitor therapy delivery.

In 1995, Masamune *et al*. [2] developed the first MRI-compatible robot, a 6-DOF stereotactic manipulator made of polyethylene terephthalate linkages and actuated with ultrasonic motors (USR30-N4, Shinsei Corporation, Japan). The robot was able to fit inside a 0.5-T closed-bore MRI scanner with 3-mm positioning accuracy inside a watermelon. Since then, various groups have developed robots for MRI-guided neurosurgical interventions.

The NeuroArm robot, one of the most prominent MRI-guided robots, has dual dexterous arms with piezoelectric actuation for stereotaxy and microsurgery [21]. The end-effector was designed to hold microscissors, bipolar forceps, suction devices, and needles. During the first 35 patient trials, one adverse event was encountered with no patient injury. NeuroArm is capable of moving a 500-g payload at 200 mm/s to a target with 1-mm positioning accuracy and 0.01° orientation accuracy [22]. However, the NeuroArm is not intended to operate during imaging. Thus, the ability to use real-time MRI to guide intervention is limited.

Though NeuroArm is primarily used for neurosurgery, it is a versatile robot but with a formidable cost. Another design philosophy is to develop compact, procedure-specific, and lower cost solutions that do not require being installed into an MRI suite. Li *et al*. [10] developed a 5-DOF cannula placement robotic assistant that is kinematically equivalent to a Leksell frame. It was based on piezoelectric actuation and allowed simultaneous imaging and intervention without impacting MR image quality [10]. Compared with the workflow of the current manual frame approach, the MRI-guided robotic approach has the potential to save substantial operating time by avoiding an additional CT imaging session with associated CT-MRI fusion and the time-consuming localization method. In terms of accuracy, the MRI-guided needle placement experiment demonstrated a three-axis rms error of 1.38 ± 0.45 mm. Li *et al*. [23] describe a newer generation of robotic assistants, primarily for MRI-guided precision conformal ablation of brain tumors using an interstitial high-intensity therapeutic ultrasound ablator probe. This robot had 8-DOF, the root-mean-square error of the tip position was 1.45 ± 0.66 mm, and orientation was 1.53 ± 0.69° during phantom studies. With regard to design, kinematic redundancy stems from the desire to lock in the instrument’s 5-DOF position and orientation and then enable insertion of a cannula, insertion of the instrument, and rotation of the instrument. The full robotic system, as described in detail in [24], was evaluated in a preclinical trial of needle-based therapeutic ultrasound (NBTU) while monitoring thermal dose live with MRTI in seven swines, as described in [25]. The results show strong agreement between MRTI and histology (mean difference in ablation volumes of 0.052 ± 0.042 cm^3^), further suggesting the ability to effectively use intraoperative MRI to guide ablation volumes using real-time thermal dosimetry from real-time intraoperative MRTI during robotic thermal therapy delivery.

There have been significant advances in MRI-guided neurosurgery for minimally invasive intracranial tumor removal. Ho *et al*. [26] first developed a brass prototype of a minimally invasive neurosurgical intracranial robot (MINIR) that was actuated by a pair of antagonistic shape memory alloy (SMA) actuators and relied on the temperature control of the SMA actuators for the control of joint motion. This work was followed by the development of a polymer-based neurosurgical robot prototype that was actuated by SMA spring actuators [27]-[29]. The new generation of MINIR robots was 3-D-printed and allowed for highly dexterous and independent control of the various segments of the MINIR robot and was tendon driven [30]. To enable real-time control of the MINIR robot, further refinement in the actuation strategy using SMA’s was achieved through active cooling of the SMA’s via a detailed modeling and experimental characterization of the actuator performance [31]-[33]. Further advancement in the control of the MINIR robot was achieved via adaptive backbone stiffening of the various segments of the robot [34]. This was followed by the development of a cable-driven, remotely actuated MINIR robot to make the MINIR robot lightweight and be able to place it on the patient’s head inside the MRI bore [35]. This design consisted of the use of a gear transmission mechanism to transmit the actuator forces to the robot end-effector and a quick-connect mechanism of easy assembly and disassembly of the robot module from the actuator and transmission system. Further refinement in the robotic system was the development of a more robust transmission system that used a combination of timing belts and gearpulley systems, as well as a switching mechanism, which eventually led to the reduction in the number of motors used, by half [36]. Furthermore, a majority of the components in this MINIR-II robot were 3-D-printed.

Guo *et al*. [37] developed a hydraulic-driven MRI-guided robot for bilateral stereotactic procedures. Originating from piezoelectric motors in the control room (master unit), the hydraulic power was transmitted via semirigid long (10 m) pipes to actuate the follower unit, which enabled intraoperative robot manipulation within the constrained imaging head coil. The stiffness coefficient of transmission could reach up to 24.35 N/mm with a 2-bar preload. The needle targeting performance was validated in a simulated deep-brain stimulation task, where the average accuracy was within 1.73 mm. Two novel MRI-based wireless tracking markers (see Section VIII-B) were adopted to offer real-time (30–40 Hz) 3-D localization of the robotic instrument under the proper MR tracking sequence. The MRI compatibility test was also conducted, where the SNR loss was within 3% even during full motion of the robot.

One MRI-guided robotic device for neurosurgery that received FDA clearance is the Monteris stereotactic platform (Minnetonka, MN, USA), which is an MRI-based system for minimally invasive laser interstitial thermal therapy (LITT). Physicians can remotely control the NeuroBlate laser probe (1-DOF translation and 1-DOF rotation) driven by a 2-DOF piezoelectric motor actuated robotic device. The laser can be planned and controlled via the computer workstation under MR thermography guidance. Other products are coming close to commercialization for more dexterous, compact piezoelectrically actuated MRI-guided robots for stereotactic neurosurgery within the MRI bore. Such systems have the potential to improve outcomes for patients undergoing a variety of neurosurgery procedures, including functional brain disorders (e.g., DBS for Parkinson’s and epilepsy), glioblastoma treatment with real-time MRI-based thermal dose monitoring, and other stereotactic procedures. MRI-compatible neurosurgery robots that can operate inside of the MRI scanner with synchronous imaging may offer improved accuracy through the use of intraoperative soft-tissue imaging, offer the potential to minimize errors, and reduce procedure time.

Future advancements in MRI-guided neurosurgery will require, in addition to the previously mentioned challenges (see Section II) of operating in the MRI environment, significant attention to design choices to make the robot highly dexterous, compact, and lightweight, if it is to be mounted on the patient’s head and maneuvered to the target location inside the brain. Furthermore, with the advancements in 3-D printing technology, it is not impossible to envision a fully 3-D-printed robot that can be patient-specific [38] not only for the specified operational workspace inside the brain but also have a customized robotic actuation system to enable the required amount of end-effector forces and torques specific to the procedure and the patient anatomy.

### C. MRI-Guided Robots for Breast Interventions

Mammography X-ray is the current standard for breast cancer detection, but it has variable sensitivity, especially in dense breasts, and imposes ionizing radiation. Furthermore, mammogram imaging can only detect the shape and density of lesions, not the tissue characteristics. MRI is sensitive to cancer tissue that may be difficult to detect with mammography, ultrasound, or palpation.

The first robotic system for biopsy and therapy of breast lesions was introduced by Kaiser *et al*. [39] in 2000. They reported a robotic system that enabled simultaneous imaging and intervention in the isocenter of the magnet. *In vitro* experiments were performed in two pig livers containing eight capsules of vitamin E, and all eight capsules were hit precisely.

Chan *et al*. [40] developed an MRI-guided manipulator driven by piezoelectric motors for needle-based interventions in breast tissue image-guided automated robots (IGARs). In a phantom model, it demonstrated an accuracy of 0.34 mm and a repeatability of 0.2 mm. In a clinical human trial, it successfully targeted a lesion and placed all of the tools for breast biopsy.

**Fig. 3.**
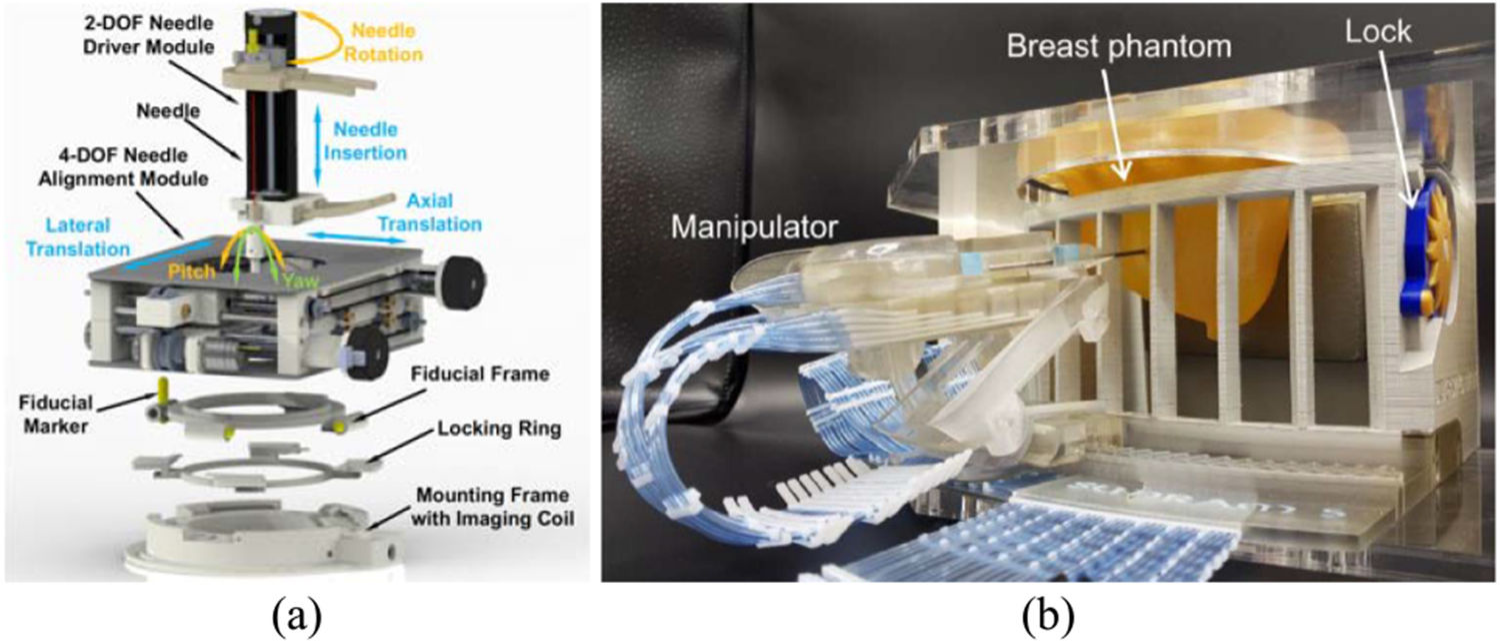
(a) CAD model of the 6-DOF body-mounted robot, demonstrating the major components: the 2-DOF needle drive module, the 4-DOF needle alignment module, the fiducial frame with fiducial markers, the locking ring, and the mounting mechanism with an integrated imaging coil [49]. (b) Sunram 5 targeted a breast phantom inside the fixation system [194].

Groenhuis *et al*. [41] designed four generations of Stormram, an MRI-compatible needle manipulator with four DOF to conduct breast biopsies in the MRI scanner. The system was driven by two linear and two curved pneumatic stepper motors. The robot can be controlled in the manual control mode and the automatic mode. Experiments showed that the mean positioning error was 0.7 mm (reproducibility = 0.1 mm). Velocity measurements with 5-m-long tubes showed a maximum stepping frequency of 8 (maximum force)–30 Hz (unloaded). The newest generation of their robotic system for breast biopsy is the Sunram 5 robot, as shown in Fig. 3(b). It has 5-DOF actuated by six pneumatic stepper motors. In the lateral direction, the actuators yield a step size of 0.3 mm, and in the vertical direction, the rotational joints have a step size of 0.3°, which translates to 0.52-mm displacement. Submillimeter accuracy was achieved based on the step sizes [194].

One of the key challenges in developing MRI-compatible robotic systems for breast biopsy and RF ablation (RFA) of breast tumors is the need to provide the clinician with the ability to remotely target the lesion with high accuracy and minimize sampling errors. Furthermore, it would be further beneficial to remotely sense the tool-tissue interaction forces. These requirements would necessitate teleoperated robotic systems with the ability to provide force feedback to the operator. Such a teleoperated robotic system will need to satisfy the severe limitations of hardware that can be deployed inside the MRI bore and in the vicinity of the MRI. While several approaches have been developed to actuate robots inside the MRI, the pneumatic actuation of the robotic system is challenging due to long pneumatic transmission lines from the air pressure source to the robot inside the MRI room. Yang *et al*. [42] presented a detailed analysis of the design and control of a single DOF robotic system that was driven by pneumatic actuation using long pneumatic transmission lines. In this study, a detailed valve model and the transmission line model are presented, and three separate controllers were designed and evaluated for precise position control. Based on the original design of Tan *et al*. [43], which involved the detailed design, analysis, and fabrication of an MRI-compatible triaxial fiberoptic force sensor, the force sensor integrated with the needle driver was also evaluated in this study [42].

Yang *et al*. [44] developed a teleoperated robotic system that was evaluated in *ex vivo* studies and targeting accuracy for *in vivo* swine studies. In the MRI-compatibility study to evaluate the patient-side robot, the loss in SNR ratio was minimal (less than 8% variation) throughout the operation of the robotic system that involved both the pneumatic actuation of the system and the piezoelectric motor actuation to perform needle insertion. Both *ex vivo* and *in vivo* targeting studies were also performed. The peak force of needle insertion into the tissue was slightly over 6 N.

### D. MRI-Guided Robots for Orthopedic Interventions

For many years, low back pain (LBP) has been both the leading cause of days lost from work and the leading indication for medical rehabilitation [45]. In developed countries, more than 70% of adults experience LBP with 20%–70% experiencing recurrent symptoms [46]. If physical therapy and nonnarcotic pain medications fail to relieve the patient’s pain, further workup is warranted. If imaging shows degenerative facet disease, the next step in diagnosis and treatment of LBP commonly involves an image-guided diagnostic and therapeutic facet joint steroid injection [47].

Current perineural injection procedures involve the use of fluoroscopy or CT to provide needle guidance, resulting in ionizing radiation exposure to the patient and physician. In addition, fluoroscopy and CT provide limited visualization of peripheral nerve anatomy, which may result in suboptimal needle placement and failed procedures. Although ultrasound is free of ionizing radiation, it is user-dependent, and good nerve visualization can be technically difficult, especially for nerves in and around the bony structures of the spine and deep nerves, such as those in the pelvis. Our research groups at the Children’s National Hospital and the Johns Hopkins University have developed an MRI-compatible body-mounted robot to allow interventional radiologists to position and manipulate the needle, while the patient is in the bore of the magnet [48], [49], as shown in Fig. 3(a).

The 6-DOF robot is composed of a 4-DOF needle alignment module and a 2-DOF remotely actuated needle driver module, which together provide a fully actuated manipulator that can operate inside the scanner bore during imaging. The system minimizes the need to move the patient in and out of the scanner during a procedure and, thus, may shorten the procedure time and streamline the clinical workflow. The robot is devised with a compact and lightweight structure that can be attached directly to the patient’s lower back via straps. This approach minimizes the effect of patient motion by allowing the robot to move with the patient. The robot is integrated with an image-based surgical planning module. A dedicated clinical workflow is proposed for robot-assisted lower back pain injections under real-time MRI guidance. The targeting accuracy of the system was evaluated with a real-time MRI-guided phantom study, demonstrating the mean absolute errors of the tip position to be 1.50 ± 0.68 mm and the needle angle to be 1.56 ± 0.93°. An initial cadaver study was performed to validate the feasibility of the clinical workflow, indicating the maximum error of the position to be less than 1.90 mm and the angle to be less than 3.14° [49].

### E. MRI-Guided Robots for Cardiac Interventions

Robotic catheters capable of remote-controlled navigation for intravascular cardiac procedures have been developed, including commercial robotic catheter systems by Stereotaxis (Niobe^1^ ES Magnetic Navigation System 2012), Hansen Medical (Sensei^1^ X Robotic Catheter System 2012), Catheter Robotics [185], and Corindus [186]. These systems rely on X-ray fluoroscopy, supplemented with electroanatomical mapping and navigation systems (CARTO^1^ 3 System—Advanced 3D Cardiac Mapping 2017, EnSite Precision^2^ Cardiac Mapping System 2017, and Rhythmia HDx^2^ Mapping System 2017 [187]), for intraoperative guidance. In these systems, the robotic catheters are controlled remotely through teleoperation. Therefore, the operator is no longer subjected to ionizing radiation from fluoroscopy imaging.

Although X-ray fluoroscopy can produce visualizations of the vascular anatomy with high spatial and temporal resolutions for procedures to be performed in easily discernible blood vessels, it does not allow soft-tissue visualization needed for performing other intracardiac procedures, such as ablation [188]. Intraoperative MRI, with its flexible visualization and greater soft-tissue contrast over intraoperative X-ray fluoroscopy, transesophageal echocardiography (TEE), and intracardiac echography (ICE), has been proposed as a superior alternative for performing cardiac catheter procedures (e.g., [82] and [190]).

One of the primary challenges in realizing MRI-guided robotic catheters is the MRI-compatible actuation of the catheter. Several research groups developed robotic catheter systems for intravascular cardiac procedures, including designs with tendon-driven (e.g., [51]-[54]), SMA (e.g., [55]-[57]), hydraulic actuation [58], and pneumatic actuation. The Hamlyn Centre for Robotic Surgery proposed a novel teleoperated robotic platform that provides an intuitive user interface and clinical familiar workflow [195], [196]. The patient-side robot was capable of manipulating conventional catheters and guidewires in multimodal imaging environments. This manipulator was produced by additive manufacturing and using pneumatic actuation. Magnetic actuation is an alternative technology used in active catheters, to avoid challenges associated with friction, backlash, and limited bandwidth associated with the abovementioned catheter actuation technologies. The main advantage of magnetic actuation is that the steering torques are generated directly at the catheter tip, rather than transmitted mechanically through the catheter from outside the patient’s body, thus increasing the bandwidth by reducing backlash and friction. However, frequently used magnetic actuation approaches using a permanent magnet (e.g., [59] and [60]) or a ferromagnetic object (e.g., [61] and [62]) are not suitable for use inside MRI. A more recent MRI-compatible magnetic actuation technology relies on microcoils embedded in the catheter body for actuation [63]-[67].

Lee *et al*. [68] designed a hydraulic-driven robotic platform to assist intracardiac catheterization, particularly, cardiac electrophysiological (EP) intervention. Such a procedure is an effective solution to treat arrhythmia. Utilizing the leader–follower hydraulic actuation units, multiple DOFs of motion can be transmitted to manipulate the robot in the MRI scanning room. With a tailor-made catheter holder, a standard EP catheter can be tightly mounted on the robot to achieve teleoperated rotation, bending, coarse, and fine insertion. The system was validated to have small (1.29 mm at 0.1 Hz) hysteresis and quick response (66 ms at 15 Hz), even at a high preloaded fluid pressure (0.2 mPa). To enhance the performance of the platform, kinematic model-based and model-free control methods were tested [69], and a continuous motor was integrated to accomplish long-range insertion [70]. Recently, utilizing the 3-D curvature reconstruction provided by multicore fiber Bragg grating (FBG) techniques, a learning-based modeling method is developed and implemented on this robotic platform to achieve feedback control of the cardiac catheter [191].

Another important challenge in realizing MRI-guided robotic intracardiac catheter procedures is that iMRI is inherently slow, making true real-time imaging challenging. Although they are improving, MRI techniques used for cardiac procedures are currently limited to approximately 7–12 frames/s [71], [72], which is not fast enough to ensure appropriate positioning of the catheter throughout the cardiac cycle via robotic control. Although there are approaches to further decrease scan time [73], they are not suitable for real-time imaging due to long reconstruction times [74]. The development of techniques for real-time iMRI for intraoperative image guidance is an active area of research.

### F. MRI-Guided Robots for Oral Interventions

Transoral laser microsurgery is a solution to treat early-stage glottic cancers since the highly localized laser beam enables precise tissue incision and minimal thermal spread to the surrounding area. Such operations also benefit from MRI during the ablation with the real-time thermal distribution of the 3-D resection margin evaluated. Fang *et al*. [75] proposed an MRI-guided soft robotic manipulator for MRI-guided transoral laser microsurgery. To comply with and pass through the constrained oral cavity, the robot was designed in miniature size (Ø12 × 100 mm) with two segments (i.e., an active bending segment for coarse robot navigation and a fine distal laser manipulator). Both segments were separately composed of three elastomeric chambers, which can be adjusted by microvolume (<0.004 mL) fluidic inflation/deflation. The hydraulic power was transmitted by leader–follower units. To resolve the nonlinearity of robot actuation, a learning-based model was trained and validated using path-following ablation tasks (mean error < 0.20 mm). Accredited to the polymer-based materials and hydraulic actuation, no image artifacts were observed in the MRI compatibility test, even during robot operation. MR thermometry successfully provided intro-operative thermal diffusion monitoring in a cadaveric head-and-neck trial.

## III. ACTUATORS FOR MRI-GUIDED ROBOTICS

Actuator design is a key challenge for MRI-compatible robots because actuators and drive electronics are often the primary sources of MRI compatibility issues. Moreover, actuator design dominates the structure and dimensions of a robot to ensure that a robot can fit inside the constrained space of a closed-bore MRI scanner. Typical robot actuators, such as dc motors, are contraindicated due to their magnets, coils of wire, and ferrous enclosures. The most common actuator technologies used in MRI robots are pneumatics, hydraulics, and piezoelectrics. Some groups have attempted to develop MRI-powered actuation techniques, such as [76], but these are not within the scope of this review.

### A. Pneumatic and Hydraulic Actuation for MRI-Guided Robots

Fluid-driven actuators (e.g., pressurized air and liquid flow) can be designed with nonactive energy sources inside the MRI room to minimize EMI. A typical configuration is to connect a robot (placed in the MRI room) with its control box (placed in the control room) through long transmission air hoses. Unlike electric actuation (e.g., piezoelectric motors) that operate on electrical energy in the MRI room, pneumatic and hydraulic actuation can operate on fluids inside the MRI room; thus, it does not compromise imaging by passing electrical noise into the scanner room [184]. Typically, hospitals have highly filtered and dried medical-grade compressed air available to use.

A major challenge of servocontrolled pneumatic actuation is accurate position control. The compressibility of pneumatics results in limited torque/force output and low stiffness. Friction force and long pneumatic transmission hoses (typically 10 m long) typically result in slow response and more than millimeter-level positioning error. Yang *et al*. [42] developed a sliding mode controller to compensate for the low bandwidth and its position error, which demonstrated 2.5–5.0-mm accuracy to reach targets inside phantom models. This approach was utilized by Fischer *et al*. [77] and Tokuda *et al*. [78] to control a pneumatically actuated robotic needle guide for prostate biopsy. This system incorporated the use of pneumatically operated brakes to lock the system into position to increase stiffness and eliminate jitter in the position controller once aligned. It should be noted that pneumatic cylinders can be controlled with both pressure control and flow control valves. When using pressure control valves, one also can readily control applied force—this can be particularly useful for providing haptic feedback on a teleoperated controlled robot in the MRI room, such as described in Shang *et al*. [79] at the Worcester Polytechnic Institute (WPI).

An alternative to servocontrolled cylinders is stepping actuators. Pneumatic stepping motors that do not require closed-loop control were studied to overcome position control challenges. Stoianovici *et al*. [80] developed the first pneumatic stepper motor and custom fiber optic incremental encoder. This motor was utilized in a robot for transrectal prostate biopsies [81].

In contrast to pneumatic actuation, hydraulic actuators, operated with incompressible liquid (e.g., water and oil), offer more accurate and responsive mechanical transmission than pneumatics. Hydraulic actuation typically requires addressing liquid leakage issues (e.g., setting up a robot may cause liquid leakage as the process requires disconnection and reconnection of the hydraulic hoses through a waveguide between the MRI room and control room). Guo *et al*. [37] presented a hydraulically actuated robot for bilateral stereotactic neurosurgery. Recent advances in sealing methods (e.g., rolling diaphragm) offer more reliable and clean solutions to avoid liquid leakage. While not within the scope of this article, hydraulic and pneumatic actuation have a foothold among MRI-compatible manipulators for biomechanics and neuroscience studies due to the ability to provide high force magnitudes with good force reflection and precise timing while synchronized with fMRI acquisition.

### B. Piezoelectric Actuation for MRI-Guided Robots

Piezoelectric motors are favorable for MRI-compatible applications due to their high accuracy, fast response times, and compactness. Since piezoelectric motors operate on the reverse piezoelectric effect, they generate no magnetic field, as opposed to traditional electromagnetic motors. Broadly, there are two categories of piezoelectric motors–ones that run off of harmonic and nonharmonic driving signals. Harmonic motors typically operate with high voltage sinusoidal drive waveforms at or near the motor’s resonance. Nonharmonic motors often have legs that walk along a surface using several lower voltage arbitrary waveforms. Regardless of the category, commercially available piezoelectric motors and motor drivers typically cause interference within the scanner bore when used without special design considerations as evaluated in [82] and [83]. Harmonic motors (e.g., Nanomotion Ltd., Israel) are generally driven with a fixed frequency sinusoidal signal on two channels at 38–50 kHz, and velocity control is through amplitude modulation of 80–300-V supply. Shinsei motors (Shinsei Corporation, Japan), also driven by harmonic signals, can be speed controlled through frequency modulation.

However, commercially available piezoelectric motors and motor drivers typically introduce up to 40%–80% signal loss during synchronous robot motion. The electrical shield with RF shielding cloth and grounding of the shielded control cables still resulted in an 80% SNR reduction. The motor driving signal is typically the source of noise instead of the motor itself, as evaluated by Fischer *et al*. [83]. Carvalho *et al*. [84] at WPI showed that, with appropriate control electronics and replacement of the metal housing of a motor, such actuators can be used with minimal electrical noise or paramagnetic artifacts induced.

Taking MRI compatibility into account from the beginning in designing a robot controller has shown to be very effective at reducing the noise and image degradation to undetectable or insignificant levels. A well-shielded control box with low noise power supplies, filtered electrical signals, fiber optic communication, and custom motor drive electronics designed to produce clean waveforms has been demonstrated to provide excellent MRI compatibility for piezoelectric actuation [18], [23], [24], [183], [201]–[203]. Such an approach can be effective at driving both nonharmonic piezoelectric motors, such as the four-channel PiezoLegs that require relatively low-voltage arbitrary waveforms, and harmonic motors, such as the two-channel Shinsei that requires higher voltage sinusoidal waveforms. Su *et al*. [85] described a piezoelectric motor driver with signals generated from a direct digital synthesizer. In contrast to commercial drivers based on high frequency switching voltage regulators, this driver is capable of generating signals for both harmonic (e.g., Shinsei and Nanomotion) and nonharmonic (e.g., Piezo Legs) commercially available motors with more detail about the control approach in [86].

## IV. MATERIALS, MECHANICAL DESIGN, AND MECHANISMS

### A. Materials for MRI-Guided Robots

As mentioned in Section I-B, the high magnetic field within the MRI environment, RF signals interaction, and switching gradients are major challenges in developing MRI equipment. MRI safe/conditional materials, actuators, encoders, and other sensors are key elements of robotic systems for MRI applications. The limitations on material selection pose a unique challenge for MRI robots that must be compact and stiff while avoiding many typically used materials and components (e.g., bearings, linear guides and lead screws, gears, and other hardware) in robotic manipulators.

Ferromagnetic materials should be avoided inside an MRI room, not only because they could cause image artifacts and magnetic field distortion but more importantly because they can pose a dangerous projectile risk to the patient and medical team [87]. Nonferromagnetic metals (e.g., aluminum, brass, and titanium), high strength plastics (e.g., PEEK [88] and ULTEM [14]), 3-D-printed (e.g., ABS and ULTEM [89]) or laser-cut (e.g., acrylic [90]) plastics, and composite materials (e.g., fiberglass [91]) are compatible with the MRI field. However, the use of conductive materials close to the scanner’s isocenter must be limited because of the possible Eddy currents that could be induced during scanning procedures, deforming the magnetic field homogeneity, creating noise and artifacts to MR images, and inducing vibration, which can manifest itself as a motion artifact in the MR images. Moreover, eddy currents could heat up the conductive materials, posing a danger to the patient [86]. Therefore, all cables that could come in direct contact with a patient’s skin should be covered. More generally, the electrical systems must be properly shielded and filtered to limit noise emission and heating. All materials that come in direct contact with the patient’s tissue or needles (e.g., a needle guide) should be constructed from certified biocompatible materials (ISO-10993).

A major challenge faced by MR-guided interventions is the lack of effective compatible instruments. This is especially true for catheter-based procedures, where polymer only-based guidewires have lower bending stiffness compared to their metallic counterparts. This led to the development of not only new fiber composites, utilizing both glass and aramid fibers (Kevlar^1^), but also new manufacturing technologies based on micropultrusion, i.e., micropullwinding. To prevent RF-induced heating in selective catheters, researchers explored substituting metal braids with nonmetallic, nonconductive polymeric braids, such as aramid fibers in selective catheters. In line with robotic endovascular interventions, a design was proposed, where two catheters, each with its own single deflectable segment, are placed over one another, referred to as “concentric or telescoping catheters.” This method not only improves controllability but also increases the number of DOFs of the catheter [193].

### B. Mechanical Design, Manufacturing, and Mechanism

MRI-guided robots can be classified as the table- and body-mounted systems based on the mounting approach [48], [92]. Table-mounted robots are fixed to the scanner bed, and for preserving targeting accuracy, the patient typically is intended to remain still during the procedure. Table-mounted robotic systems that could be heavier and more rigid are suitable for certain interventions, such as prostate biopsy (see [11], [90], and [82], [93], [94]), stereotactic neurosurgery (see [30], [95], [96], and [182]), and long bone biopsy [4]. Nonetheless, patient motion is inevitable, especially for procedures that require a longer time. Therefore, mechanical fixtures, such as the Leksell frame utilized in stereotactic neurosurgery, are usually used to restrict patient motion. An example of such fixation is described in the MRI-compatible stereotactic neurosurgery robot developed by Li *et al*. [23].

On the other hand, body-mounted robots are attached to the patient directly (e.g., using straps or other methods) to attenuate the errors related to the patient’s motion by moving with him/her. Because dedicated supporting frames are not required, body-mounted robots must be compact, lightweight, and easily attachable to the patient body [92]. Body-mounted robotic systems have been used for MRI-guided renal cancer interventions [97], abdominal interventions [98], [99], shoulder arthrography [100], [18], and lower back percutaneous [48] procedures.

Available fabrication methods for MRI-guided robot components depend on the material type. Generally, the nonferromagnetic metals [18] and high strength plastics, such as PEEK [88] and Ultem (or polyetherimide) [14], [93], are precisely cut using conventional machining. For other plastic materials, such as Delrin and ABS, the fabrication opportunities vary with the type of plastic, and fabrication type is a significant factor in the performance of the robot components [101]. Delrin (or Acetal) has high MRI compatibility and can be precisely cut by water jet cutter or other CNC machining procedures. A more cost-effective alternative is to use ABS, which could provide complex 3-D structures produced in rapid prototyping easily, but that does not guarantee better mechanical performances [102].

The main drawback associated with many of the materials used in MRI-guided robotic systems is their limited structural stiffness, which can have a negative effect on the manipulability and accuracy of robotic devices [3]. To address these issues, different teams, considering the clinical application, developed a wide range of mechanical serial (e.g., [82]) and parallel (e.g., [94], [18], and [48]) architectures. Furthermore, choosing a particular actuation method and the actuator location in an MRI-compatible manipulator is of paramount importance and will affect the entire design of the system [104]. Three main strategies have been observed in the literature: 1) generating motion using traditional EM actuators (or any other types of non-MRI-compatible actuators) located outside the scanner room [105]; 2) using piezoceramic actuators (see Section III-C) inside the scanner room but located at a distance from the scanner isocenter to ensure MRI compatibility [106]; and 3) placing the actuators (commercially available, such as in [92], or custom-made, such as in [93]) inside or close to the scanner isocenter, such as in [4]. Each of these solutions has advantages and disadvantages that must be carefully considered for specific clinical applications.

## V. SENSING AND LOCALIZATION FOR MRI-GUIDED ROBOTICS

Realizing precise sensing, localization, and tracking for the position of interventional devices and tissue anatomy under MRI is significant for clinical diagnosis and intervention. MRI markers have improved the accuracy of positioning and control for surgical procedures, including the operations of neurosurgery, biopsy, renal denervation, brachytherapy, and so on. In addition, MRI markers can help to implement the prescription or surgical plan with automatic 3-D positioning and motion compensation. Unlike electromagnetic tracking methods, such as inertial measurement units (IMUs), FBGs are not affected by many surrounding effects, including water immersion and electromagnetic fields, which could be practically suitable for surgical MR suites and without effects on image quality.

Precise tracking for the interventional instrument in MRI allows positional feedback and realizing closed-loop control for surgical robotic systems, as well as enabling the instrument position to be visualized in the control room overlaid on imaging [107]. The development of MRI-compatible tracking technologies has improved the accuracy of localization and interventional instruments, such as insertion needles and catheters in real time. Some tracking methods, including fiber optic sensors and integrated tracking coils, show potential for surgical robots because of their sensing performance in MRI. Also as noted earlier, image-based tracking can serve as primary or redundant means of localization and confirmation of the robot’s instrument placement.

### A. Proprioception Sensing: Fiberoptic Shape Sensors

Placement of needles and other needle-like devices is a fundamental application in interventional MRI though there are multiple challenges depending on the target anatomy and clinical procedure. A review of MRI-guided needle-based interventions can be found in [87]. The basic problem is to place the needle tip at a location specified on an MRI image volume, going through an entry point (very likely at the skin surface), also specified on the images [108]. Robot-assisted needle placement in the brain was one of the first uses in interventional MRI, and this technique has since been extended to other parts of the body, including prostate, breast, liver, spine, and so on [2], [10], [20], [109]-[111], [192]. There is extensive literature on MRI-guided robotic systems for needle placement. This section will discuss some common aspects of needle localization in MRI, real-time needle tip localization and shape detection, and adaptive needle guidance.

For correct manipulation of needles, catheters, and other flexible tools inside the patient’s body, it is important to track their 3-D position and shape during a surgical procedure in real time. Considering the complex morphologies of anatomical targets, tortuous path access, constrained space requirements, and inherent tool deformability, shape reconstruction is still very challenging. Instrument localization and shape detection in MRI can come from a number of means, including image-based, integrated tracking coils, or sensorized instruments.

Recently, optical fiber technology, specifically FBG, has gained attention for curvature estimation and shape reconstruction [112]. The FBG-based shape sensors can be built using multiple (the most widely used configuration is triangular with three outer fibers) single-core fibers [113]-[115] or multicore fibers, having several cores integrated into a single fiber [116]. An increased number of cores will provide better accuracy of the shape sensor at the same core spacing [117]. Besides shape detection, the FBG-based sensorized tools could be calibrated in force for simultaneously detecting the interaction forces between the instrument and surrounding anatomy [116]. Among the FBG-based sensorized instruments, better performances in shape reconstruction have been provided by the systems that undergo small deformations (e.g., steerable needle for biopsy and ablation) [112].

The FBG fiber is a sensing technology that works by detecting the optical signal difference induced by mechanical or temperature strain change. The incident spectrum emitted from the light source passes through the gratings, where most of it can transmit, and the remaining spectrum is reflected. Such a system can be used to measure needle deflection, determine the 3-D shape of a needle, or assess forces acting on a needle.

The FBGs’ shape sensor has a high-frequency capability of shape estimation in real time. For example, an MRI actuator using an FBGs’ sensor developed by Moerman *et al*. [118] could reach more than 100-Hz high capture rate under 50-N force load. This sensor system allowed a maximum error of 0.043 N [118]. In addition, the feature of excellent multiplexing capabilities with thin and submillimeter diameters enables the writing of a series of sensing gratings on one fiber without changing the fiber diameter [119]. Park *et al*. [120] demonstrated a small-gauge biopsy needle with 18 Ga × 15 cm inner dimension using FBG sensors to estimate bending deflection for the needle and provide temperature compensation. Besides, the high flexibility of the optical fiber allows it to be integrated with delicate or soft devices with a minimal effect on the instrument stiffness. Wang *et al*. [121] developed a soft robot with an FBG fiber helically wrapped on the surface to measure the motion data (torsion, curvature, and lateral force) for a manipulator in minimally invasive treatment. In the research of Mandal *et al*. [122], a registration pipeline with three FBGs was proposed to the sensor and gets the centerline of the vessel for reconstructing the catheter in real time.

These distinctive advantages of FBG fibers have prompted their employment in many applications, including shape sensing of steerable interventional needles [123], [124], navigation of medical instruments [122], force sensing of surgical devices [125], [200], and temperature sensing for surgical devices in MR environment. Temperature monitoring using FBGs’ sensor is able to provide an adequate spatial resolution for minimally invasive treatment. Some researchers have developed temperature sensor systems by FBGs for kidney and pancreatic tissue treatment in animals (rabbits and swine); such FBGs’ sensing systems allowed for observing temperature change of hyperthermia treatment under MRI [126]-[129].

The wavelength-division multiplexing (WDM) and the optical frequency-domain reflectometry (OFDR) are the most widely common options in wavelength modulations for optic fiber shape sensors. The main difference is that WDM can only integrate a limited number of FBGs on one fiber at a low cost [130], [131]. OFDR technology is used to develop all grating fibers by analyzing the optic signal in the frequency domain so that it can enhance the accuracy of tracking and can read thousands of FBGs on a single fiber of a 100 m length. Fiber with multicore FBGs could further improve the sensing density and enable 3-D shape sensing of the fiber configuration [133]. Francois *et al*. [134] used the OFDR sensor to track the 3-D shape of the catheter (3-DOF for the fibers) and provided the positions of the surgical instruments in real time with a submillimeter accuracy for MRI-guided interventions.

Although the FBGs have distinctive capabilities of sensing in many applications, the FBGs rely on spectrum analyzing technology, and the commercial cost of optical spectrum analyzers is expensive, which may increase the cost of the high precision measurement in real time. In addition, the FBGs may not allow the high resolution for the detection of microstrain. These drawbacks still remain challenges to wider adoption of the FBGs technique.

Needle insertion under MRI guidance has been proposed for interventions such as biopsy, cryoablation, and injections [87]. To reduce tissue damage and avoid areas, such as vital organs, asymmetric bevel-tipped flexible needles can be adaptively guided by needle steering. Besides the real-time shape sensing, planning the needle insertions requires accurate models for the future shape and needle-tip position prediction. Starting with the groundwork of unicycle/bicycle models for needle shape determination [166], [167], [167], flexible needle steering models have included varying kinematics-based models [168] and mechanics-based models [169], [170]. The former has been used in the control and planning of the needle but may not properly capture additional or complex needle deformation. The latter models are usually based on classical beam theory to determine needle shape but require detailed information on the tissue. As an alternative, a sensor-based model using a Lie-group theoretic approach has been proven successful for shape sensing in multilayer tissue using FBG sensors [30]. This model, which is based on the theory of elastic rods and Lie groups, has the advantage of complex needle shape recognition in 3-D space through the use of FBG sensors. Furthermore, this model has been extended to accommodate needle shape prediction in single-layer and multilayer tissues [171].

### B. Exteroception Sensing: Tracking Coils

Instruments, robot end effectors, and registration fiducials can be localized in MRI using tracking coils, often as microcoils integrated into the device that either creates a signal to be picked up by the imager’s receive coils or acts as a receiver coil themselves. The tracking coils are typically composed of several loops of carrying-current wire, capacitors, and peripheral electronic components (e.g., resistor and photodiode) [135]. Such circuits can either be used for producing a magnetic waveform at a particular frequency or picking out a signal from a bunch of signals [136]. In the MR environment, the protons that absorb energy at a specific frequency will reemit energy in the form of magnetic waveforms at the same frequency [137]. When clusters of local waveforms pass through the closed-loop coil and change magnetic flux, the coil circuit resonates and then transmits amplified signals, which creates a high imaging contrast, thus allowing the position tracking for MRI-guided interventional control [138].

The MRI tracking coils can be broadly classified into three types, including passive [139]-[141], active [156]-[158], and semiactive tracking coils [142]-[144] based on the approaches used in the procedures of RF generating and signal receiving. The active tracking coils are wired by conductive cables to the separate receiving channels, while the MR system could activate the small coils by RF pulse and then selectively pick up resonating signals around these coils to contract images [145]. Unlike the active tracking coils, the passive coils neither need to carry target-oriented RF nor require external hardware or wired connection with the MRI. The received MR signal is amplified by the coils to enhance the imaging contrast of the interventional robots and immediate vicinity [146]. It remains a matter of controversy to classify the semiactive coil. On the one hand, it serves for passive visualization because the markers are not linked with the receive channels. On the other hand, the coils within the MR control are in general electrically active [143]. Here, we unify the hybrid coils as the semiactive type.

Rigid devices often can be visualized using the susceptibility artifact. The optical tracking system using an external referencing system could be used in cases where automated tracking is needed. For nonrigid instruments (e.g., for endovascular procedures), the passive visualization based on the susceptibility artifact of the device and the active tracking system using microreceive coils could be useful [204]. Zimmermann *et al*. [205] used a small inductively coupled marker coil attached to the chest wall and detected with a fast projection technique for quantification of the respiratory motion of the thoracic wall. Volunteer studies with the marker coil showed a good agreement with simultaneously acquired breathing belt data and position information extracted from the MR images [205].

#### 1) Passive Tracking Coils:

The passive tracking coils are generally divided into two categories: wired [147]-[149] and wireless coils [145], [151], [152]. The wired coils driven by the external controlled direct current (dc) can induce magnetic field distortions, which results in the local susceptibility artifact for instrument visualization [145]. However, it is now rarely used owing to the RF heating effect [153]. It should be noted that the dc-driven coil tracking is perceived as a passive method although it is electrically active. The main reason is that the self-contained tracking is completely independent of the MR system. Here, we concentrate on wireless coil-based passive tracking. These wireless markers are essentially resonant *LC* circuits that are composed of amalgamated electronic components (e.g., RF coils and nonmagnetic capacitors) [152]. They do not need any elongated conductors to carry the RF signal. Instead, they receive signals and amplify the flip angle (FA) during the RF excitation process. Relying on the inductive coupling effect, the amplified signals are captured by the commercial transmitter/receiver MRI coils, which enhances imaging contrast [145]. Apart from the amplification of amplitude, it is critical to align the resonant frequency (*f_m_*) with the Larmor frequency (*f_L_*) of MR systems in the frequency domain. Referring to [155], *f_m_* can be formulated as

(1) $$f_{m}=\frac{1}{2\pi \sqrt{LC}}$$

where *L* is the marker inductance and *C* is the capacitance. It indicates that the resonate frequency is in inverse proportion to the product of capacitance and inductance. Furthermore, effective amplification of the MR signal depends on the markers’ quality factor

(2) $$Q_{m}=\frac{1}{R}\sqrt{\frac{L}{C}}=\frac{2\pi f_{L}L}{R}.$$

The ratio of inductance *L* and resistance *R* linearly determines the quality factor, which provides a theoretical guide for the marker miniaturization design. Thus, these potential miniaturization methods, including increasing turn numbers and decreasing the outer size of the inductor or conductor diameter, could lead to a higher *Q_m_* [151].

Overall, the wireless coil-based tracking method transcends the limitation of the wired connection and then eliminates the concerned heating issue [139]. The miniaturization achieved by the advanced fabrication process bestows the substantive integration degree on coils. However, this passive technique may be limited by orientation dependency. Some studies concentrated on single-wound solenoid-shaped coils found that the amplified MR signal might be lost [145], [151]. Moreover, the rigid mechanical properties of the passive markers make them not applicable to some critical parts of the body (e.g., the cerebral cortex) [152].

#### 2) Active Tracking Coils:

The active tracking approach mostly utilizes wired coil units that are linked with the separate receiving channels through conductive coaxial cables [145]. Beginning with transmitting a sequence of RF pulses originating from the MR system, then the coils actively sample signals along with the three orthogonal directions. Their respective position information could be computed from the RF pulse sequences during projection readouts [156]. Another study showed that the coil-based active tracking method achieved a spatial resolution of 0.6 × 0.6 × 0.6 mm^3^ in real time (40 Hz) [157]. In short, the advent and development of active coil-based active tracking constitute a significant step toward fully MRI-guided clinical interventions. An example of such an application is the tracking of an NBTU ablation probe for the above-described stereotactic neurosurgery robot using a pair of active tracing coils to locate the tip position and the axis of the instrument [24]. Compared with passive coil tracking, it enables higher spatial [140], [141] and temporal [158], [159] resolutions, which potentially shortens procedure times and improves surgical outcomes. However, the active tracking coils still exhibit substantial shortcomings in the aspects of safety and steerability. More specifically, the elongated conductive wires would act as an RF antenna to generate heat that may hurt the surgical robots or damage the tissues of patients [159]-[161]. One previous study discovered that the maximum temperature was upward of 74 °C after 30-s scanning [162]. Other researchers intended to address the heating problem by adding transformers to the conductive transmission lines [163] or introducing quarter-wavelength coaxial chokes [164]. However, these measures, in turn, further complicate the coil integration with surgical instruments. Furthermore, both the active and passive coil tracking methods only achieve finite steerability in tortuous and narrow parts (e.g., blood vessels) of the body attributed to its rigid mechanical structure.

#### 3) Semiactive Tracking Coils:

Recent studies on coil-based semiactive tracking are seldom reported. In semiactive tracking, it is relevant to note that these coils have no direct electrical connection with the MR system although they are physically linked to each other. These markers are stand-alone *LC* circuits that resonate at the MR Larmor frequencies [145]. As with the passive coils, the amplified signals by semiactive coils are also inductively coupled to MRI receiving coils. Its unique feature is that the electrical characteristics can be tuned by external triggers within the charge of the MR system [165]. For instance, the MR sequence controlled an optical laser signal source to manage optical transport in an optical fiber affixed to a catheter [151]. The light finally illuminated a photodiode sensor in a resonating circuit, which was placed at the catheter tip point. The drop in the resistance of the photodiode could change the resonant parameters of the semiactive tracker, thus turning it into the “detuning” status. The status switch between “detune” and normal resonating minimizes the artifacts in case of motion, which offers the robustness of the tracking [143]. In sum, the coil-based semiactive tracking solves the heating issue by benefiting from the replacement of conductors (e.g., optical fiber). Moreover, the hybrid approach reaches higher robustness than both the passive and active coil tracking techniques. Conversely, the demand for a more complex hardware setup and more precise synchronization control hinders its promotion in MR.

The three main methods of coil-based tracking could provide positional feedback to enable accurate manipulation in diverse MRI-guided application scenarios. These coils exhibit their advantages and shortcomings in the aspects of positioning accuracy, imaging contrast, efficiency, the complexity of hardware setup, and MR sequence design, safety, and miniaturization. State-of-the-art coil tracking techniques are summarized in Table 1.

## VI. SYSTEM ARCHITECTURE

When developing a robotic system to work in an MR environment, there are a number of design decisions to take into account. One key decision is whether the system is tightly coupled to a particular MRI suite installation, or if the system is readily set up and taken down in an arbitrary scanner as needed. Tight integration with a particular scanner suite can offer some advantages; one such advantage is that application-specific cabling can be integrated into the suite through custom penetration panels. Furthermore, larger more permanent installations of controllers, robots, user interfaces, and the like may be set up and left in place. Examples of such systems include the teleoperated NeuroArm [21] and the pneumatically controlled Innomotion [11] robots. However, a significant downside to integrating with the MRI suite and having large, fixed equipment is that it: 1) requires custom installation at the hospital adding significantly to the cost and complexity of use and 2) the system is tied to a particular location. Some robots intended to operate in the MRI environment are developed with the specific intent to not require any customization to the scanner suite and can be readily set up in any scanner quickly. To pass signals in and out of the scanner room to the console area and/or the equipment room, electrical signals should be passed through filtered connectors built into the penetration panels through the room’s shield, and nonelectric signals, such as fiber optic cables, pneumatics, and hydraulics, should pass through the standard waveguide tubes that penetrate the shielded walls. While passing electrical wires through a waveguide is convenient and may be possible for some aspects of development, the wires act as antennas and bring noise into the scanner room; thus, electrical signals should not be passed through waveguides. Often, the penetration panels of MRI rooms have very limited connections, and it can be challenging to have these customized for a particular system in most cases. Therefore, the architectures that operate most effectively are: 1) the robot should have a control system designed such that it can be operated from within the scanner room such that the only signal passing through a preexisting waveguide is a fiber optic communication cable or 2) all signals passing in/out of the MRI room are fiber optic, pneumatic, and/or hydraulic that are routed through the waveguide to a control system in the console area or equipment room. An example of an in-room electronic control system is [23] and [85], and an example of an external rack coupled to the robot via nonelectronic connections would be [173].

Further aspects of system architecture hinge on integrating the various required subsystems. In general, such systems comprise the robot, the robot controller, the planning and navigation software, the iMRI system, and, optionally, a separate imaging server and other therapy delivery and/or monitoring equipment. One technique for integrating the various subsystems is to put them all on the same network (in some cases, even on the same computer) and communicate via a standardized communication protocol, such as OpenIGTLink [174]. Some MRI-guided robotic systems can operate with intraoperative interactively updated imaging, in this case, a standard DICOM push, or other means may be used to transfer images from the MRI scanner to the planning and navigation platform for the robotic system. However, to most fully leverage the advantages of MRI-guided robotic systems, one would want to receive live imaging from the scanner and use that to control positioning and/or therapy delivery with real-time feedback. Furthermore, live imaging may be used for closed-loop feedback for positioning, such as in [20], or therapy delivery, such as real-time thermal dose maps from MRTI to guide thermal ablation as described in [175]. Most scanner vendors have means for streaming images out in real time to an external computer, and furthermore, they have means for using an external system to control the scan geometry and other parameters on the fly (e.g., automatically changing scan plan position and orientation to match the robot pose predicted by the kinematics or localized fiducials). This also becomes important for monitoring active and passive tracking coils or otherwise tracking instruments intraoperatively in the MRI scanner. However, such interfaces are often proprietary and specific to each vendor (and, sometimes, different scanners from the same vendor), so there is a challenge in creating devices that can readily operate on a variety of MRI scanner manufacturers and models. There have been some efforts to develop software middleware based on OpenIGTLink [174] to wrap the vendor-specific API and expose standardized interfaces to the robot control software.

## VII. REGISTRATION AND PLANNING

### A. Robotic System Registration

To relate a target position in the patient coordinate system, which is often coincident with the imager’s coordinate system, to that of the robot, one must perform a registration step. This allows finding the robot in the MRI scanner such that, after a target is identified, the robot’s kinematics can be used to plan a trajectory to get there. Image to robot registration is an essential step to knowing how to command the robot to reach a target in image space. The result of the registration step is typically a coordinate transformation matrix relating the coordinate system of the medical images to the coordinate system of the robotic system. Registration techniques for MRI robotics are often based on rigid registration methods, which were originally developed for image-guided interventions. Typically, four or more MRI-visible fiducial markers are placed on the robot frame for registration purposes. These markers are identified in the MRI image and used to relate the MRI coordinate system to the robot coordinate system. In some cases, a 3-D volume is acquired, and the fiducial markers located in the imaging are related to the known fiducial configuration on the robot using a technique such as least squares. Alternatively, unique patterns of fiducials can provide a full 6-DOF registration from a single crosssectional slice. An automatic fiducial frame detection and registration method for device-to-image registration in MRI-guided prostate interventions was presented in [176].

Such fiducials may be passive markers (such as capsules of MRI contrast agent), or they may be tracking coils as described above. A 6-DOF hybrid tracking method, comprising passive fiducial tracking for initial registration and subsequent incremental motion measurements with MRI-compatible joint encoding, was presented in [14]. The objective was to develop an alternative tracking methodology for an MRI-guided transrectal system for prostate interventions with the following performance goals: 1) measure 6-DOF pose with accuracy comparable to or surpassing previously reported approaches; 2) employ only standard MRI pulse sequences; 3) minimize embedded electronics; and 4) MRI-compatibility with no imaging artifacts. While there are many techniques for registration, it is beyond the scope of this article to address all approaches for robot registration and registration between image sets.

### B. Surgical Planning

When using an image-guided robot, optimizing the clinical workflow and user interface is critical. Surgical planning is an essential step to allow the clinician to identify the target anatomy and plan a path from the skin entry point to the target. This can be done directly on the intraoperative images, or it may be done on preoperative images and then registered to the patient during a procedure. Furthermore, live iMRI during a procedure enables monitoring and updating the surgical plan intraoperatively.

For example, in MRI robotics for needle-based interventions, a slicer-based planning interface was created for neurosurgery applications by Patel *et al*. [92]. This planning interface allows the clinician to identify target and entry points in the imaging, and display the intended robot trajectory overlaid on intraoperative images. This approach is representative of many such systems. Automated planning algorithms are a work in progress.

A custom-developed planning program was written for the MRI-guided prostate interventions system [14]. Similarly, with the above-presented example, the planning interface displays the acquired MRI images, provides the automatic segmentation for the initial registration, allows the physician to manually select the targets, provides targeting parameters for the needle-tip placement, and tracks the needle position provided by the encoders, while the manipulator is moved on target and logs data.

## VIII. REAL-TIME MRI-GUIDED FEEDBACK CONTROL AND SURGICAL INTERVENTION

### A. Real-Time Feedback and MRI-Based Thermal Monitoring

As noted in Section II, MRI offers the ability to monitor and track anatomy, instruments, and therapy delivery in real time. This can be applied in a number of ways, including tracking the path of a needle as it is inserted (either assessing unintended deformation or tracking a steerable needle), assessing target motion (such as brain shift or respiratory motion), and monitoring dose delivery (such as thermal dose during ablation).

While the advantages listed for using MRI include high spatial resolution and real-time imaging, it should be noted that there are tradeoffs that one must make between image quality, the field of view, and update speeds. This becomes an important design decision when implementing real-time monitoring, where, in some cases, it may be advantageous to trade off image quality (often in the form of lower SNR or spatial resolution) for update rate. It is possible to create custom MRI scan sequences to optimize the speed of accurately localizing a needle. Taking into account the robot, initial guesses of needle location can be derived from the registration and robot kinematics to further speed up the update rate and increase the reliability of image processing for needle localization. In one such example, Wartenberg *et al*. [20] described tracking a needle and target in real time and using this to automatically compensate for errors in the needle path. The system has force sensors on the robot, and the speed of insertion (along the intended path) is controlled through a cooperative hands-on teleoperation control scheme. However, as an asymmetrically tipped needle is inserted, it is automatically rotated by the robot to steer the tip toward the target (based on real-time MR image feedback), much like an automated lane following a vehicle.

In addition to tracking the instrument and the anatomy, MRI offers the unique ability to track the temperature and from that calculate a live 3-D thermal dose map (typically calculated as CEM43). Such an approach enables real-time control of the ablation boundary. For the above-described neurosurgery robot, Gandomi *et al*. [175] describe modeling the thermal dose of a rotating directional NBTU applicator to perform conformal ablation. Closed-loop control of arbitrarily shaped lesions based on real-time MRTI-based dosimetry in phantoms has been demonstrated, and preclinical trials are ongoing. MRI offers tremendous potential for utilizing real-time temperature measurements to generate live thermal dose maps, which, when coupled with robot control of an ablator (e.g., NBTU, HIFU, LITT, Cryoablation, RFA, and MWA), offers the potential for closed-loop conformal ablation to ensure complete coverage within the margins while minimizing collateral damage to nearby sensitive tissue.

### B. Coil Tracking for Closed-Loop Position Control

Achieving real-time and precise position tracking of interventional robots and patient anatomy plays a significant role in MRI-guided intervention and diagnosis. Because of this, the position feedback could realize closed-loop control of surgical robots thus improving the clinical outcomes [75]. Coil-based tracking is one promising method that has been widely studied and employed in MRI-guided interventional applications.

**Fig. 4.**
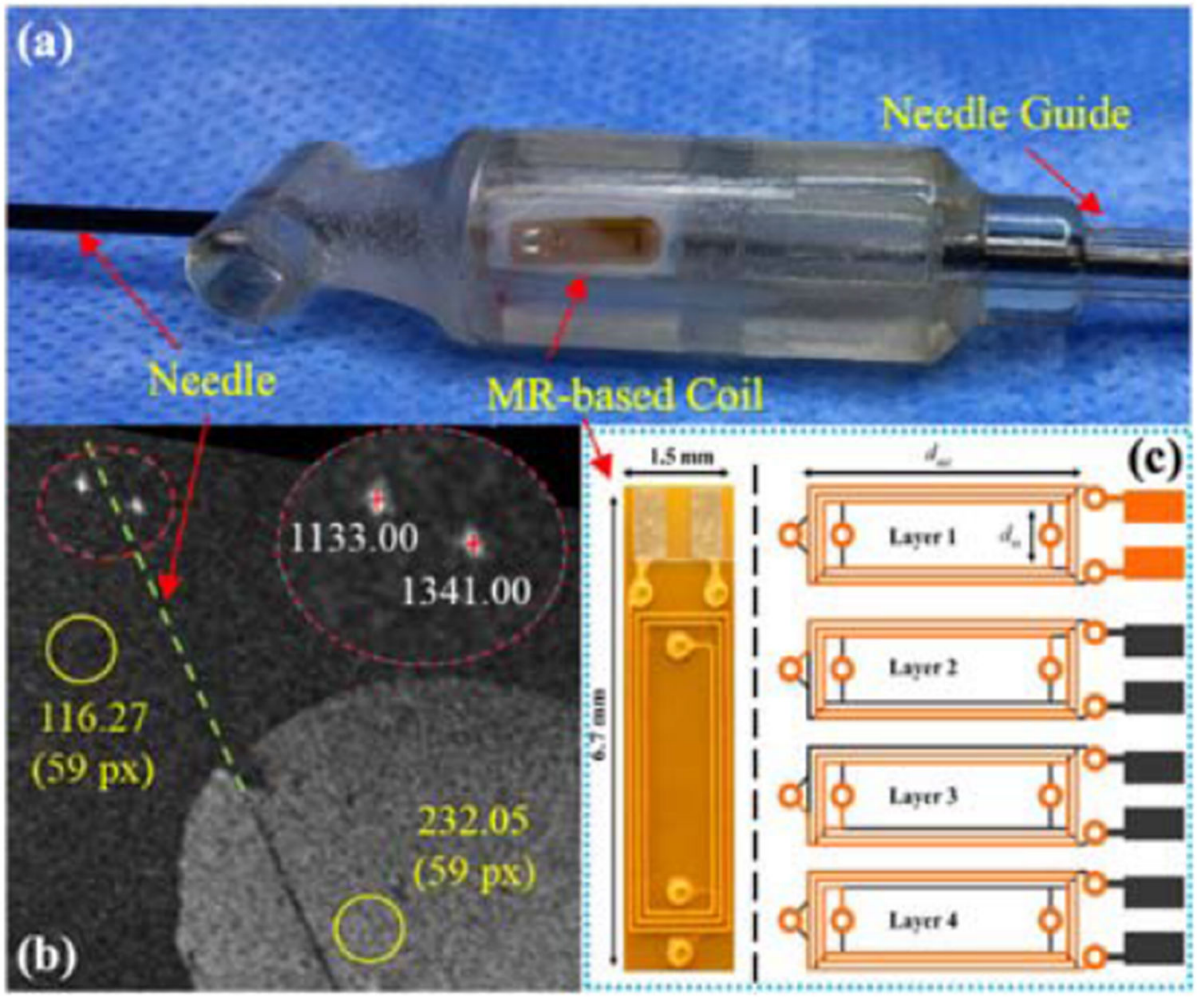
(a) Needle guide contained two coil units, and one was shown on its sidewall [37]. (b) Two bright spots indicated the coil position in the MRI. The high signal intensities at two red crosses formed a sharp contrast to the intensities of two yellow circular areas [37]. (c) Real prototype of a four-layer coil with outer dimensions: 1.5 mm × 6.7 mm [145].

Two small-size films of MRI-compatible coils (1.5 × 5 × 0.2 mm^3^) integrated within the needle guide [see Fig. 4(a)] were first used to achieve position tracking for MRI-guided bilateral stereotactic neurosurgery [37]. The study showed that both the needle and coils could be visualized in MRI. The amplified intensities of their center points rose to 1341.00 and 1133.00, which formed high imaging contrast to the average intensities of the agar-gel brain and background area [see Fig. 4(b)]. On account of the reliable and real-time (30~40 Hz) position feedback, the experiment reached a high degree of accuracy in MRI-guided robot control. Within a 48 mm radius, the maximum position error was 0.50 mm with an inherent precision error of 0.12 mm. Fig. 4(c) showed a real prototype of a wireless coil made of four-layer flexible printed circuits (FPCs). The two rectangular copper pads on the top side eased the replacement of the capacitor, which contributed to the adjustment of resonating frequency. In another study, Dai *et al*. [177] demonstrated the feasibility of coil-based tracking to control and navigate the robot platform in an MRI-guided ultrasound system. In this design, wireless RF coils (6.7 mm × 1.5 mm) were attached to three tiny cylindrical glass tubes to amplify the local MR signal. These tubes were filled with gadolinium-doped fluids (concentration: 10 mM). The background signal can be eliminated to shorten the water relaxation time by introducing CuSO_4_ solution into the MRI-compatible hydraulic actuator. The robot position information obtained from three markers was used to register with the MRI coordinate system. There were three peak points along with the 1-D projected axis, whose position values could be extracted from the intensity profile. The result proved coil tracking provided real-time positional feedback at a high refresh rate of 83.3 Hz.

## IX. DISCUSSION

A wide variety of robots for MRI-guided interventions have been developed to overcome the challenges of MRI compatibility requirements. Most of those robots are procedure-specific, i.e., they are designed for one specific type or class of surgical intervention. Some early research resulted in advanced, more general-purpose MRI-compatible robotic systems. Hashizume *et al*. [178] presented an MRI-guided robot for minimally invasive laparoscopic surgery. This system was provided with both laparoscopic imaging and MRI; thus, it enabled visualization of both exterior and interior tissues. The robot successfully performed punctures to reach a 2-cm-diameter target on three pigs with MRI-guided laparoscopy. Though this type of versatile robot (e.g., [178] and NeuroArm) might be more advantageous in some regards, the constrained space within the MRI bore and the cost are both design considerations that potentially impede clinical adoption of a versatile MRI-guided robot [107].

Since MRI can be more expensive as compared to other imaging modalities, minimizing the time of the procedure in the MRI room is critical for the realization of this technology. The iMRI time decreases with the increasing adoption of scanners with higher strength fields, along with the increase in the spatial resolution of the scanner and the imaging quality. As technology continues to advance, the idea of the digital OR, which could include robotic systems, comes closer to realization. MRI robots could be part of the digital platform by enabling ionizing radiation-free procedures with interactive imaging, superior soft-tissue visualization, and therapy delivery monitoring. Also, there are opportunities to further explore the potential benefit of MRI-guided robots through the application of emerging technology, such as autonomous control. FBGs are not affected by many surrounding effects, including water immersion and electromagnetic fields, which could be practically suitable for surgical MR suites without sacrificing image quality and, thus, provide an ideal way of shape sensing inside MRI.

Pediatrics is an ideal area for MRI-compatible robotics since MRI is typically widely available in pediatric hospitals. At most pediatric hospitals, there is widespread consensus that exposure to ionizing radiation for medical purposes should be minimized or eliminated whenever possible. The “Image Gently” campaign from The Alliance for Radiation Safety in Pediatric Imaging, which promotes dose reduction, was launched in 2008 [50] and has had widespread momentum. By moving pain injections from CT to MRI, we can provide exquisite soft-tissue visualization while eliminating radiation exposure.

To make MRI-compatible robots broadly applicable, it is important to minimize the restrictions on where they can operate. While some hospitals have specialized interventional MRI suites, there is a major benefit to developing robots that are capable of operating in the large installed base of high-quality diagnostic MRI scanners. Thus, there is a commercial benefit to building compact, application-specific, scanner vendor, and MRI room configuration agnostic robotic systems. There could be great benefit to having robots that can be readily and quickly placed onto the bed of standard diagnostic MRI scanners without compromising workflow and that do not require customization of the imaging suites.

Since most MRI-guided robots operate at least in part with some aspect of teleoperation rather than fully automated procedures, real-time iMRI is crucial for visual feedback to clinicians. Since diagnostic MRI systems are generally not designed for interventional imaging, there is a strong need to address latency issues of MRI images for human-in-the-loop teleoperated control. Many scanner vendors do offer a means for rapid streaming of imaging (and remote control of the scanner). The approaches are ideal when real-time imaging is used as part of an automated closed-loop system. However, an alternative that does not require any special interfaces is to use iteratively updated imaging wherein the scanner can push images to a planning software each time a volume is acquired—this can be very effective for applications where iterative or incremental motion is acceptable. Thus, it is critical to identify the needs of a particular clinical application to determine the required update rates of imaging and acceptable latency for those images to be utilized in external software.

MRI-compatible coil tracking techniques have been demonstrated in fulfilling progressive tracking and seamless integration. One critical challenge is to combine tiny tracking coils with high-quality factors in interventional instruments [151], [164], in which the small size of interventional tools limits the design of coils. Another is that the shortfall of orientation dependency would result in the failure of signal amplification when the normal line of the inductor surface aligns to the magnetic field [151], [152], [179]. In the future, the development of tracking methods will focus on the miniaturization of MRI tracking devices. An additional tracking device(s) attached to a surgical interventional robot inevitably would change its physical properties (e.g., size, torque transmission ratio, and bending performance). Miniaturization could increase the usability of tools, thus allowing the better capability of performing surgery accurately. In addition, the geometry of tacking coils should be enriched to accommodate the nonrigid body transformation since the fixed geometrical layout is not feasible for nonrigid applications (e.g., catheters).

The FBGs’ sensing focuses on estimating the 3-D profile of targets, and the coil-based tracking excels at point measurement [180]. Both of them have been used extensively to provide position feedback under the MR environment. These locating devices made of glass fibers, bronze, aluminum, and plastic are intrinsically compatible with MRI [181], which showcases distinct advantages compared with traditional tacking instruments (e.g., camera, electronic–magnetic tracker, and IMU). The cumulative efforts on the two tracking approaches realize accurate locating of intervention robots in real time. However, neither can fulfill seamless integration with surgical tools due to the axial stiffness of FBG sensors and coil circuits. In particular, the small size of interventional tools limits the design of coils with high-quality factors, complicating the assembly procedure. Besides, the expensive optical spectrum analyzers on which FBGs sensors depend inevitably increase the cost of shape sensing. In the future, we believe that the development of tracking methods should focus on the miniaturization of sensors to ease the integration and then increase the usability of tools, thus enhancing the capability of performing surgery in narrow parts of the body. In addition to applications requiring large deformation, the softness and flexibility of the sensor(s) could be primarily optimized by geometrical enrichment or material improvement.

## X. CONCLUSION

In summary, there is tremendous potential for incorporating robots into MRI-guided surgical procedures. This approach offers the ability to accurately target specific anatomy that is readily visible in MRI that may not be recognizable otherwise and to be able to use that information intraoperatively. However, with that opportunity comes many challenges. With careful consideration of the workflow and other requirements, the appropriate design tradeoffs can be made to develop successful MRI-compatible robotic systems as having been demonstrated in the examples above. In most cases, the robots are compact application-specific devices intended to operate within the scanner bore during imaging. Actuation approaches must be thoughtfully assessed and incorporated into the designs, and as necessary, sensing approaches enable tracking of the robot and instrument during the intervention. The ability to perform procedures faster and more accurately utilizing intraoperative feedback endows MRI-compatible robotics with a tremendous amount of potential to transform the future of image-guided interventions.

## Figures and Tables

**Fig. 1.**
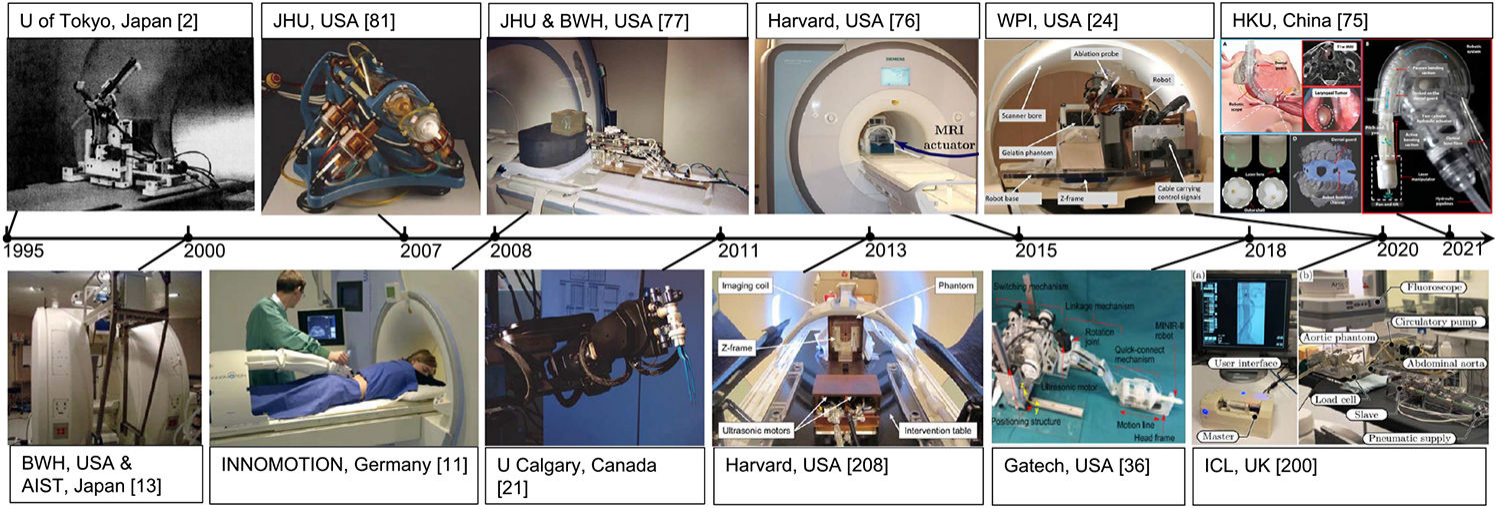
Representative MRI-guided robot-assisted surgery and interventional systems, showing a variety of different clinical applications, sensing and actuation techniques, control architectures, and mechanism designs throughout the years.

**Table 1:** Techniques of Coil-Based Tracking in MRI-Guided Interventions

	Mechanism	Advantages	Disadvantages
**Passive coil**	♦ Amplify the local FOV signal.	Simple hardware and safe integration. Adopt different field-strength MR.	Time-consumable. Low MRI resolution.
**Active coil**	♦ Use MRI sequence to generate local MR gradient magnetic field within the targeted area. ♦ Receive the MR gradient signal by the coil in the frequency domain.	♦ High temporal and spatial 3D resolution.	♦ Potential risk of RF heating issue caused hy the conductive wires. ♦ Complicated matching circuit in the receiving interface.
**Semi-active coil**	♦ Isolated LC coil controlled by MR resonates at Larmor frequency.	♦ No conductive link. ♦ No heat issues. ♦ Low artifact.	♦ Difficult to integrate and minimize the hardware. ♦ Sophisticated MR sequence design for control.

## Footnotes

1 Registered trademark.

2 Trademarked.
